# Supplementary material for: Validation of Six Commercial Antibodies for the Detection of Heterologous and Endogenous TRPM8 Ion Channel Expression
Source: Int J Mol Sci. 2022 Dec 18;23(24):16164. doi: 10.3390/ijms232416164 (PMC9784522; doi:10.3390/ijms232416164)
Supplement: Supplementary file 1 [file ijms-23-16164-s001.zip › ijms-2099118-supplementary.pdf]

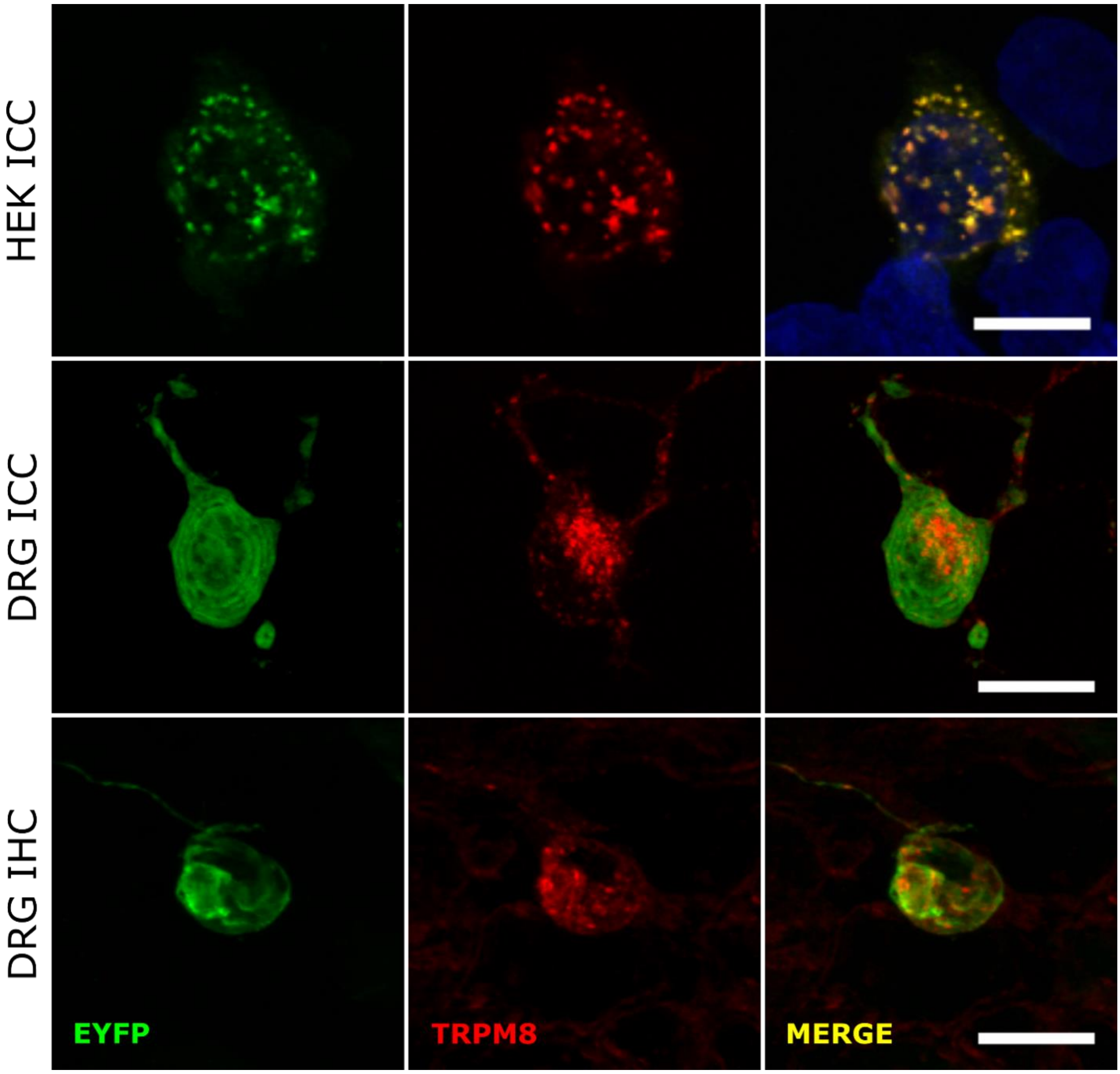

FIGURE S1. **Subcellular distribution of TRPM8 immunostaining in mTRPM8-EYFP transfected HEK cells ICC and DRG ICC and IHC.** Confocal images showing the fluorescent pattern of TRPM8 staining. Green: EYFP. Red: TRPM8 (Origene1 antibody). Blue: Hoechst. Scale bar: 10μm for HEK ICC, 15 μm for DRG ICC and IHC.

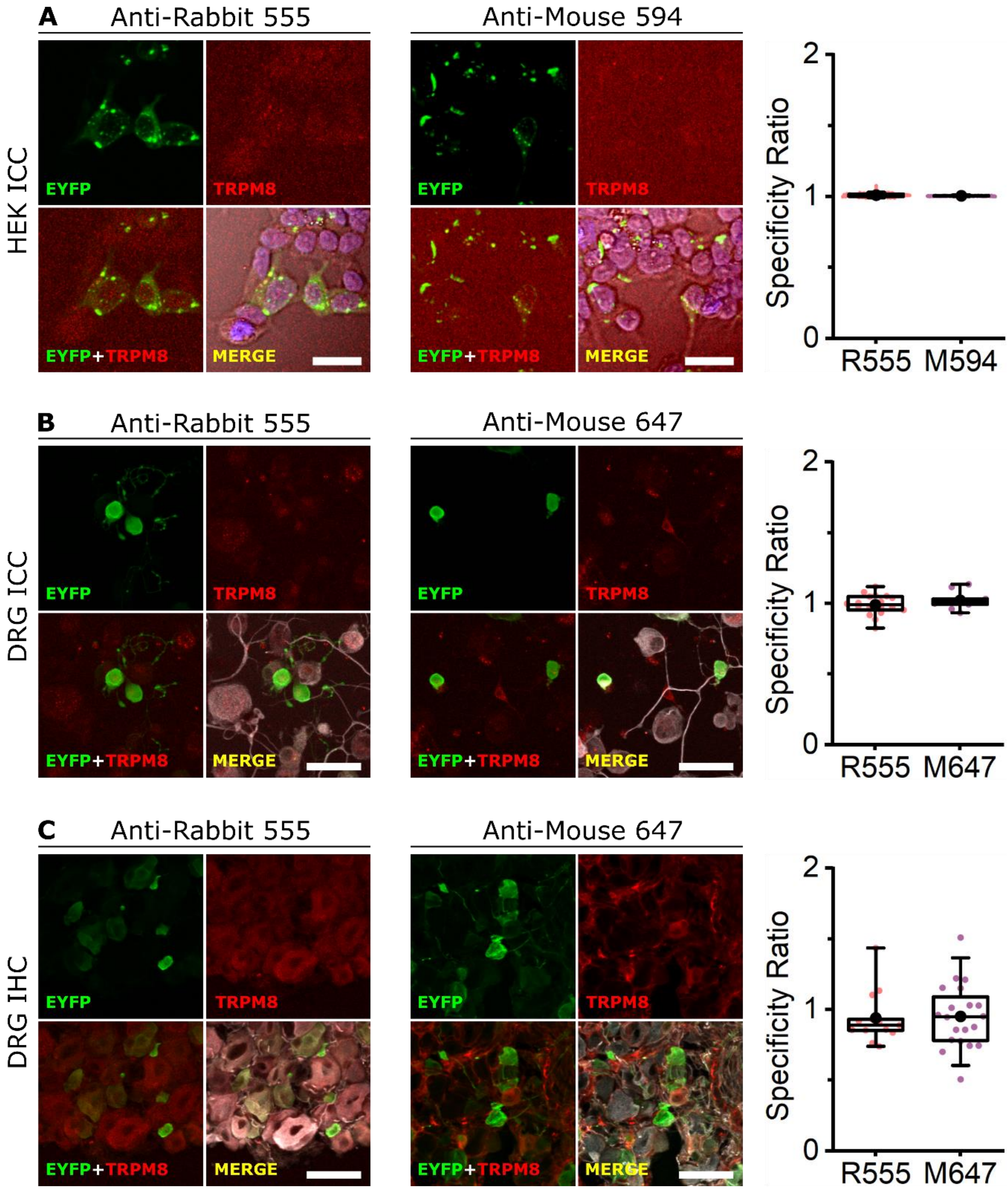

FIGURE S2. **Control immunostainings without primary antibodies. A, B, C** left, middle. Confocal images of immunochemistry in transfected HEK-293 cells (**A**), cultured DRG neurons (**B**) and DRG slices (**C**) from the *Trpm8^{BAC}-EYFP^{+}* mouse. EYFP (green), no TRPM8 primary antibody (red), Hoechst (blue), βIII-Tubulin (grey). Merge in A correspond to the overlap of the three fluorescent signals plus the bright field image. Scale bar: 30 µm for A, 50µm for B and C. **A,B,C** right**.** Box plots represent specificity ratio (SR) for each dilution of the respective antibody. Each dot corresponds to an individual EYFP+ cell. Box contains the 25th to 75th percentiles. Whiskers mark the 5th and 95th percentiles. The line inside the box denotes the median and the black dot represents the mean. n = at least 10 cells; 2 images from 1 mouse for each secondary antibody.

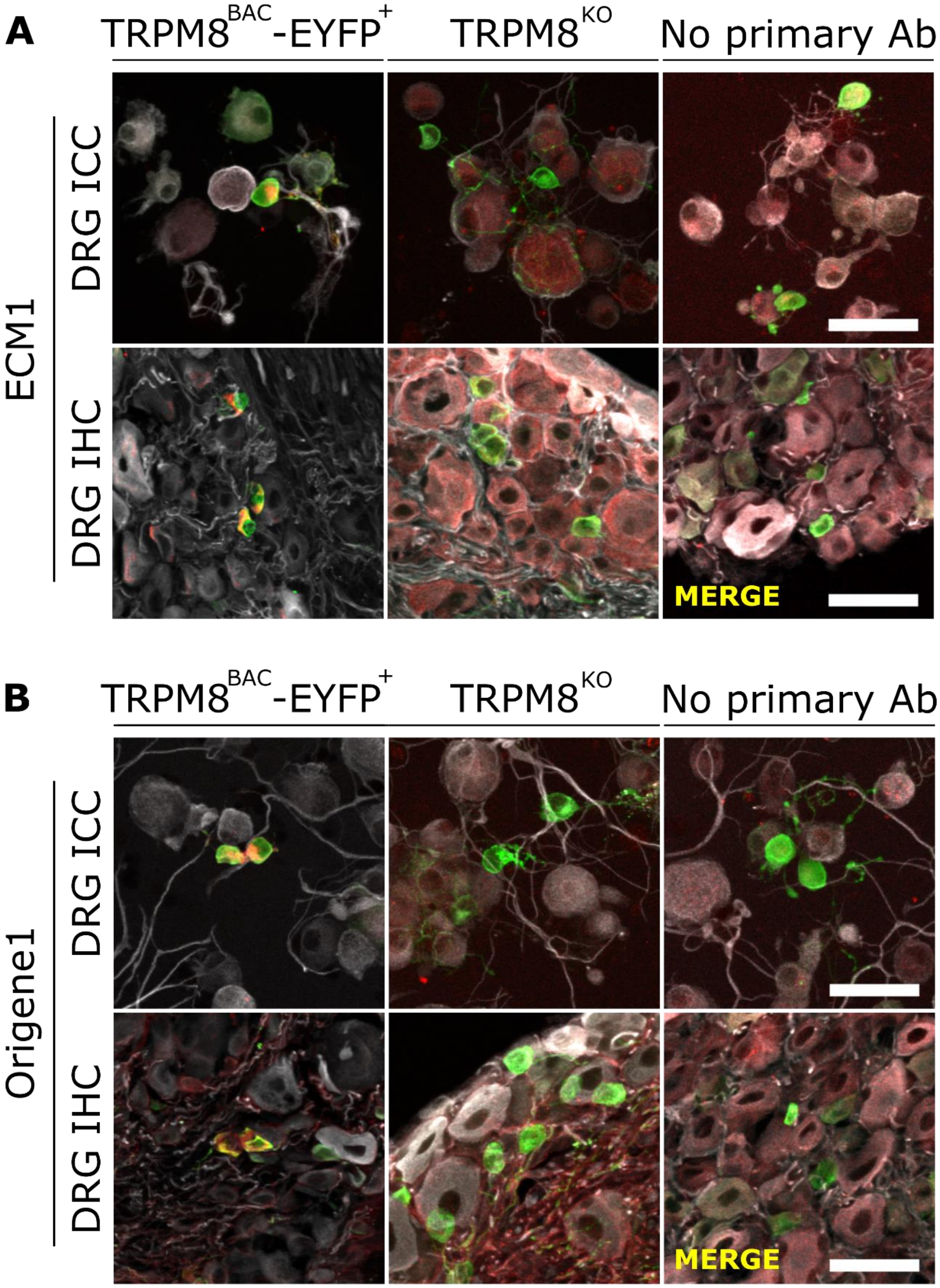

FIGURE S3 Comparison of TRPM8 immunostaining in the TRPM8 reporter mouse compared to TRPM8^{KO} mouse and to control without primary antibodies. Confocal merged images of ICC and IHC in DRGs using the ECM1 (**A**) and the Origene1 (**B)** antibodies. EYFP (green) TRPM8/secondary antibody alone (red), βIII-tubulin (grey). Scale bar: 50 µm.
